# Patients’ and physicians’ perceptions and attitudes about oral anticoagulation and atrial fibrillation: a qualitative systematic review

**DOI:** 10.1186/s12875-016-0574-0

**Published:** 2017-01-13

**Authors:** Gemma Mas Dalmau, Elisenda Sant Arderiu, María Belén Enfedaque Montes, Ivan Solà, Sandra Pequeño Saco, Pablo Alonso Coello

**Affiliations:** 1 Iberoamerican Cochrane Center, Biomedical Research Institute Sant Pau (IIB Sant Pau), Sant Antoni Ma Claret 167, 08025 Barcelona, Spain; 2 Consorci d’Atenció Primària de Salut de l’Eixample (CAPSE), CAP Casanova, Barcelona, Spain; 3 Institut Català de la Salut, SAP Litoral-esquerra, Barcelona, Spain; 4 CIBER Epidemiología y Salud Pública (CIBERESP), Barcelona, Spain

**Keywords:** Atrial fibrillation, Oral-anticoagulation therapy, Perceptions, Attitudes, Patients, Physicians, Systematic Review, Qualitative Research

## Abstract

**Background:**

Oral anticoagulant therapy reduces the risk of stroke in patients with atrial fibrillation, but many patients are still not prescribed this therapy. The causes of underuse of vitamin K antagonists oral anticoagulants are not clear but could be related, in part, to patients’ and physicians’ perceptions and attitudes towards the benefits and downsides of this treatment. The purpose of this systematic review was to evaluate and synthesize patients’ and physicians’ perceptions and attitudes towards the benefits and downsides of vitamin K antagonist, in order to explore potential factors related with its underuse.

**Methods:**

We included studies that used qualitative or mixed methods and focused on patients’ and/or physicians’ perceptions and attitudes towards oral anticoagulation. We systematically searched PubMed, EMBASE, ISI WoK, and PsycINFO from their inception until May 2013. Two reviewers independently assessed the quality of the included studies and synthesized results using a thematic analysis approach.

**Results:**

We included a total of nine studies. In four studies, the quality assessed was excellent and in five was moderate. We identified three themes that were of interest to both physicians and patients: information to reinforce anticoagulation use, balance of benefits and downsides, roles in decision-making and therapy management. Three additional themes were of interest to patients: knowledge and understanding, impact on daily life, and satisfaction with therapy. The main difficulties with the use of anticoagulant treatment according to physicians were the perceived uncertainty, need of individualised decision-making, and the feeling of delegated responsibility as their main concerns. The main factors for patients were the lack of information and understanding.

**Conclusion:**

Physicians’ and patients’ perceptions and attitudes might be potential factors in the underuse of treatment with vitamin K antagonists. Improving the quality and usability of clinical guidelines, developing tools to help with the decision-making, enhancing coordination between primary care and hospital care, and improving information provided to patients could help improve the underuse of anticoagulation.

**Electronic supplementary material:**

The online version of this article (doi:10.1186/s12875-016-0574-0) contains supplementary material, which is available to authorized users.

## Background

Atrial fibrillation or flutter is a common cardiac disorder. Estimated prevalence of patients with atrial fibrillation was 33.5 million people worldwide in 2010, of which 20.9 million were men and 12.6 million were women [1]. Incidence and prevalence are higher in developed countries and their estimated trends are growing due to population aging [1, 2]. Age-adjusted mortality in 2010 was 1.6 and 1.7 per 100,000 for men and women, respectively [1].

A high risk of stroke is associated with atrial fibrillation [3–5]. Of all patients that suffer an stroke 20–30% have atrial fibrillation [6]. Age, a history of previous embolism, heart failure, type 2 diabetes mellitus, hypertension arteriopathy, and female sex are factors that increase the risk of embolism among patients with atrial fibrillation [7, 8]. Atrial fibrillation is a health issue that is costly for the healthcare system. Costs derive mainly from hospital admissions (50%) and treatment prescription (20%) [9]. Moreover, non-treated patients experience more complications compared to treated patients leading to obvious economic consequences [10].

Vitamin K antagonists oral anticoagulants (VKAs) significantly reduce the risk of stroke in patients with atrial fibrillation [8, 11], and are the main group of drugs that has been historically used for such patients. Nevertheless, treatment with VKAs is relatively complex due to its narrow therapeutic range, which urges regular monitorization of the International Normalised Ratio to place the patient within the optimal range for anti-thrombotic protection without excessive risk of haemorrhage [6, 12]. Furthermore, VKAs show various drug interactions and, therefore, no big changes are to be made in the intake of vitamin K-rich foods. Such specificities of the treatment with VKAs are some of the potential reasons for its underuse [13–17] despite concluding evidence available on its potential net benefit [12, 18].

A new group of oral anticoagulants has recently emerged as a therapeutic alternative for patients with atrial fibrillation: the direct oral anticoagulants (DOACs). Patients taking DOACs show lower risk of stroke, intracranial bleeding, haemorrhage or death, but higher risk of gastrointestinal bleeding compared to patients receiving VKAs [19]. Additionally, patients taking DOACs show fewer interactions with other drugs and no interactions with food. However, their effect has a shorter duration, there is no known antidote and are not recommended for patients with an important kidney condition. Moreover, DOACs are considerably more expensive than VKAs, which is an essential feature to ensure equal access and treatment adherence [20]. Although the absence of laboratory monitoring for DOACs might be attractive for patient, it also entails potential disadvantages, such as the inability to measure the level of anticoagulation, determine treatment adherence, or detect potential drug interactions [21]. Nowadays, VKAs continue to be the group of drugs more frequently used in the common practice for patients with atrial fibrillation [22].

Previous studies show that preferences of patients’ with atrial fibrillation may be an important reason for underutilization of oral anticoagulation [23–25]. The previous systematic review [25] that examined experiences of patients and health providers regarding atrial fibrillation and treatment with VKAs, revealed a few factors that might be related to underutilization of VKAs. Nevertheless, there is no qualitative systematic review to date focused on knowing patients’ and physicians’ perceptions and attitudes towards VKAs that might be potential factors for underutilization of VKAs for atrial fibrillation.

The objective of this qualitative systematic review is to identify potential factors associated with underuse of VKAs oral anticoagulants. To do so, we critically synthesised the available qualitative research evidence about patients’ and physicians’ perceptions and attitudes towards this treatment alternative.

## Methods

We conducted a systematic review to synthesize findings from studies that assessed patients’ and physicians’ attitudes and perceptions of the risks, benefits, and use of vitamin K antagonists oral anticoagulants (VKAs), in order to explore the perceptions and attitudes related to the underuse of anticoagulation in patients with atrial fibrillation.

### Design

Systematic review of qualitative research.

### Data sources

We searched PubMed, EMBASE, ISI Web of Knowledge (ISI WoK) and PsycINFO from their inception until May 2013. In order to identify relevant publications, a search using a combination of key words “values or preferences”, “anticoagulants”, and “atrial fibrillation” was performed. The search strategy used is in Additional file 1.

### Study selection

Two authors independently assessed the references retrieved from the search and later resolved any disagreements. We included: i) original articles that explored the perceptions and attitudes of patients, physicians, or both, about VKAs for atrial fibrillation; ii) used qualitative or mixed methods; and iii) were published in English, Spanish, German, or French. We excluded studies that only explored perceptions and attitudes of health professionals other than physicians, and studies that did not include qualitative results.

### Critical appraisal

We assessed the quality of studies using the Critical Appraisal Skills Programme (CASP) tool for qualitative studies [26].

### Data extraction and data synthesis

We collected the main characteristics of each study included in the review (Table 1). We contacted the authors of the included studies for clarification. We used a thematic analysis to analyze the data [27]. The main and recurrent themes, as well as categories, across the studies were collected systematically. We segmented the themes and contrasted them between studies, collapsing and refining the categories per type of participant (patients and physicians) until the final result was deemed optimal.

**Table 1:** Characteristics of included studies

	Country	Methodological approach	Technique	Analysis	Quality assessment	Participants	Number (only physicians or patients)	Sex
Howitt et al. 1999 [28]	United Kingdom	Mixed: quantitative and qualitative	Structured interviews (information through email)	Thematic analysis (information through email)	moderate	-Patients	56	28 men/28 women (information through email)
Fuller et al. 2004 [32]	United Kingdom	Mixed: quantitative and qualitative	Questionnaire and interviews	Content analysis	moderate	-Patients	76 (in the interviews)	
Wild et al. 2004 [33]	United States,, United Kingdom and Spain	Qualitative	Semi-structured interview	Interpretative. Thematic analysis.	moderate	-Patients	60	
Coelho Dantas et al.2004 [35]	Canada	Qualitative	Semi-structured Interview.	Interpretative. Thematic analysis (information through email). Grounded theory.	excellent	-Patients	21	12 men/9 women
Bajorek et al. 2007, 2009 [29, 30]	Australia	Qualitative: Phenomenology	Focus groups	Thematic analysis	excellent	-Physicians	20	12 men/8 women
						[Geriatricians, Cardiologists, General medicine (consultants or registrars),General Practice] -Nurses -Pharmacist	(6 Geriatric 6 Cardiologists/General Medicine 8 General Practice)	
						-Patients	14	
						-Carers		
Solà et al. 2009 [37]	Spain	Mixed: quantitative and qualitative	Focus groups	Content analysis	excellent	Physicians [General Practice, Cardiologists and Internal Medicine]	23 (15 General Practice, 5 Cardiologist, 3 Internal Medicine)	12 men/11 women
						Patients	23	15 men/8 women
Lipman et al. 2004 [29]	United Kingdom	Qualitative	Semi-structured Interview.	Framework method	excellent	-Physicians [General Practices with an active interest in research or evidence-based medicine]	11	9 men/2 women
Anderson et al. 2006 [34]	United Kingdom	Mixed: quantitative and qualitative	Questionnaire and semi-structured interview	Interpretative. Grounded theory.	moderate	-Physicians	14	
						[Cardiologists, Geriatric or general medicine (consultants or Specialist Registrars)]	5 Cardiologists, 9 Geriatric or General medicine	
Decker et al. 2012 [36]	United States	Qualitative	Semi-structured Interview.	Content analysis	moderate	-Physicians [Cardiologists and internal medicine) -Nurses practioner	23 (18 Cardiologist 5 Internal Medicine)	

## Results

**Fig. 1:**
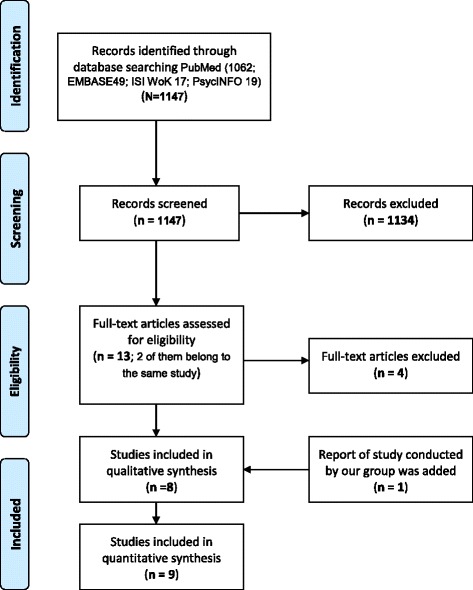
PRISMA Flowchart

We initially retrieved 1147 references. A total of 1134 publications were excluded after reading the title and abstract, and 4 were excluded after reading the full text (Fig. 1). A total of nine articles corresponding to eight studies were included [28–36]. We also included a still unpublished study conducted by our group [37]. Two of the nine studies collected data both from patients and physicians [29, 37], four collected data from patients only [28, 32, 33, 35], and three from physicians only [31, 34, 36]. In four studies, the quality assessed was excellent [29–31, 35, 37] and in five was moderate [28, 32–34, 36].

### Physicians’ perceptions and attitudes

**Fig. 2:**
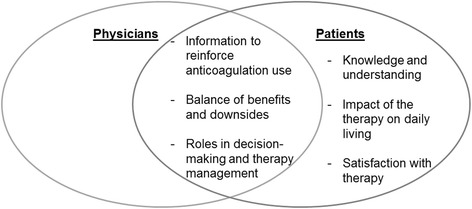
Emerging themes

The five studies with physicians as participants [29, 31, 34, 36, 37] (Table 1) included a total of 91 physicians (family physicians, cardiologists, geriatrics and internal medicine physicians). The three main themes that emerged from the analysis were: I) information to reinforce anticoagulation use; II) balance of benefits and downsides; and III) roles in decision-making and therapy management (Fig. 2).

#### Information to reinforce anticoagulation use

The information needed to reinforce the vitamin K antagonists (VKAs) use was a theme that emerged in all five studies including physicians as participants [29, 31, 34, 36, 37].

In three studies [29, 31, 37], some physicians considered the scientific evidence about VKAs was a possible barrier for the correct management of anticoagulation due to the constant changes in the literature [31]. Some expressed concerns and others justified the prescription of aspirin rather than warfarin [31]. Some referred to their lack of skills in using evidence-based medicine [31]. They also expressed concerns regarding the applicability of the evidence, which they considered did not include representative populations, and did not reflect daily practice [31]. Other comments referred to the need for more information to reinforce the decision to start the VKAs [29], the finding of ambiguities in the published clinical guidelines [31], and the importance of individualized decision-making for each patient [31, 37].

In one study [37], physicians stated that they often gave more weight to their own professional experience than to research findings for decision-making in VKAs. In relation to what information physicians should provide to patients, in one study the opinions ranged on the grade of information provided [31], although in another study there was consensus that generally the way information was given to patients was inappropriate [29] (Table 2).

**Table 2:** Physicians’ quotations

**Information to reinforce anticoagulation use**
… *although you may have*, *um*, *read things*, *eventually you stick to your experience*, *right*? *You can read that statistically the probability is low but if you have face a few cases*… *you don*’*t act in the same manner*.’ (Family doctor, Spain) [37].
. . . *if someone comes to you with atrial fibrillation you want to know*, *if he*’*s the average man in the street*, *what am I best to treat him with and that*’*s* . . .*that*’*s not answered by studies that have 80*% *exclusion rates*.” (Family physician, UK) [29].
**Balance of benefits and downsides**
“… *I believe that in the fibrillation treatment the benefits*, *I think of those moments where benefits are observed*, *regardless of the risk of the therapy in itself*…” (Family physician, Spain) [37].
“*Ideally you would want to treat this lady with warfarin but in view of the recurrent falls and the subsequent risk of life*-*threatening haemorrhage l would opt for the lesser antithrombotic of either aspirin or clopidogrel*” (Hospital family physicians, UK) [34].
**Roles in** **decision-making** **and therapy management**
“*Patients have wonderful trust in their GPs*, *which we don*’*t want to interfere with*, *but they do seem to think that the GP is going to remember and know every detail*.” (Hospital pharmacist, Australia) [29].
“…*I*’*m not so much convinced that it should be on me to decide on the indications*, *it*’*s far from clear to me* (…), *on one hand*, *and then*, *I get really angry when other specialists decide on the indication*, *which may be appropriate but they do it with no knowledge of the patient*’*s social history whatsoever*…” (Family physician, Spain) [37].
*Decision making for who goes on warfarin is taken often by one person*, *monitoring of warfarin is taken by another person and in our practice people are monitored in different systems*, *alright and er* . . . *ongoing responsibility for patient education is nonexistent* . . . *the potential risks of warfarin to me are so large in terms of errors basically*.” (Family physician, UK) [29].

Main themes are captured in bold

#### Balance of benefits and downsides

Most of the studies discussed the balance of the benefits and downsides [29, 34, 36, 37]. Physicians expressed no doubts about the benefits of VKAs [37]. In the Bajorek study [29], however, geriatricians appeared to be more focused on the risks than on the benefits. Some physicians expressed uncertainty in specific cases, such as psychiatric patients, polymedicated patients, patients that fail to attend follow up, patients at risk of falls [34, 37], the elderly, and also in certain social environments [36, 37] as alcoholic patients [37].

Some family physicians related their uncertainty in the decision-making of anticoagulation, not only to evidence but also to the experiences [37] and reactions manifested by the patients [29]. Some physicians attributed this patient negativity to the lack of adequate information [36] or opinions based on hearsay [31]. In one study [36], physicians’ main concerns about VKAs were the risk of bleeding and the International Normalized Ratio monitoring.

Only in one study [31], the physicians directly raised the topic of safety. Of note, most of the physicians did not show excessive concerns about safety. Family physicians who were especially concerned about safety described a lack of clarity in the protocols on safety and in the International Normalized Ratio monitoring. Additionally they felt that often the person ultimately responsible for the treatment was unclear (Table 2).

#### Roles in decision-making and therapy management

In all studies [29, 31, 34, 36, 37], physicians discussed decision-making. In three studies [31, 34, 37], most physicians supported shared decision-making; however, the degree of involvement varied [34]. They stated that the clearer the evidence, the less they involved patients in decision-making [34, 37]. In two studies, physicians stated that patients preferred to delegate to them [29, 37]. However, they stressed that patients themselves needed to assume responsibility for their management [29, 37]. Even some hospital physicians proposed daily International Normalized Ratio self-monitoring as the best option for the patient because it implies a more sustained control [37].

Family physicians also felt specialized physicians delegated responsibility of a complex therapy, traditionally assigned to hospital professionals [37]. Some family physicians disagreed on the indications for VKAs given by hospital physicians [29, 31, 37]. They considered this disagreement could be associated with the differences in approach to decision-making: primary care physicians support patient-centered decision-making, while other specialists are more disease-centered [31, 37]. They also recognized a lack of confidence and experience prescribing and controlling VKAs, and communication difficulties with other specialists in case of doubt, due to the lack of communication channels [37]. Some specialized physicians also recognized this lack of communication [36] and suggested that there should be more communication between primary care and hospitals to reach a consensus on the indications [37]. Some specialized physicians stated that they also considered non-clinical characteristics of the patients, such as psychosocial characteristics [36] (Table 2).

### Patients’ perceptions and attitudes

In the six studies that included patients [28–30, 32, 33, 35, 37] (Table 1) six main themes emerged (Fig. 2): I) knowledge and understanding; II) information to reinforce anticoagulation use; III) impact of the therapy on daily living; IV) balance of benefits and downsides; V) roles in decision-making and therapy management; and VI) satisfaction with therapy.

#### Knowledge and understanding

In four studies [28, 30, 33, 35] patients discussed the knowledge and understanding of VKAs, varying with patient age (it was higher among younger patients) [35], condition (lower in patients with atrial fibrillation than in those with thromboembolism), and setting (lower in patients from Spain than in patients from the United Kingdom or the United States of America) [33]. Most patients of two studies were unaware that VKAs prevents stroke [28, 30, 35] and in one particular study they did not relate with the risk of stroke nor with atrial fibrillation [30]. They did not associate International Normalized Ratio monitoring with the risk of bleeding or stroke. Only patients who had had a stroke had full knowledge of the indications for anticoagulation [30].

Patients expressed some misconceptions, hearsay [30, 33], and myths [29] about alcohol consumption, nutrition, and concurrent medication [30, 33]. These misunderstandings were likely caused by contradictory recommendations made by other patients, caregivers, and health care professionals [30]. Some reported that they had been informed that VKAs was “a sort of rat poison” [33] (Table 3).

#### Information to reinforce anticoagulation use

In four studies [29, 30, 35, 37] patients discussed the information they had received to reinforce VKAs use, with variable needs on the amount of information they wanted to receive [37]. In two studies [29, 35], some patients taking VKAs considered the amount of information received was insufficient. They felt that the information should be more detailed, especially concerning drug functioning and dose adjustments [29, 30]. They requested more information about the role and importance of VKAs, and the implications in accepting this treatment, at the time of the decision-making [29]. In another study, patients considered there was a lack of both written and verbal communication [30].

Patients also manifested difficulties in applying the knowledge during the daily management of their treatment, and felt they were “on their own” [29]. They would have preferred to receive the information gradually during follow-up and to be able to check if they had understood correctly [30], increasing their confidence in its management [29, 30] (Table 3).

**Table 3:** Patient’s quotations

**Knowledge and understanding**
*A 72*-*year*-*old male has a 30* % *chance of having a stroke regardless*, *but if I didn*’*t take the Coumadin*, *it would be a 70* % *chance of having one. So I*’*m taking medication to avoid the stroke* (Patient, Canada) [35].
*There was a sticker on one of the medication boxes that said you shouldn*’*t take aspirin with this* … *but the specialist said you take half a Solprin*™ …*so you get sort of a conflicting thing* (Patient, Australia) [30].
**Information to reinforce anticoagulation use**
*The specialist didn*’*t give me this* [*book*] … *he said that you could get this book and I had to go to two pharmacists to get one* …*I think it could be better communication* … *they just gave me the book* (*Patient*, *Australia*) [30].
*I didn*’*t get anything* … *only very sketchy in* [*hospital*]… *I haven*’*t received anything extra at all* (*Patient*, *Australia*) [30].
**Impact of the therapy on daily living**
“*I had to go get the blood drawn. It was such a pain to get the paperwork from the doctor*’*s office and go to the lab to get it drawn*, *and then you have to wait to talk to the doctor on the phone. He told you whether or not to continue it or to change the dosage or what I had to do. And then you have to go back again to see if it was the proper dose. It was a pain* (Patent, US) [33].
*I will only drink one glass of wine a day. I like a glass of wine. They say just go easy on the single malt*, *and stuff like that*…*There wasn*’*t any special* [*instructions regarding diet*]. *We like good food*, *and we eat a good*, *balanced diet. I like seafood*, *and I love fish*, *and I like the odd steak. I try to stay off butter. I*’*m taking Becel*® *just now*, *which I don*’*t really like*, *but I try to stay off the butter and cooking with all the white sauce*, *and butter sauce*, *and stuff like that* (*Patient*, *Canada*) [35] .
**Balance of benefits and downsides**
*Before anything the fact of going to the hospital so often*, *they are crammed with people*, *and you have to spend the whole afternoon there*… *just for simple shot* […] *though it*’*s a real sacrifice* (Patient, Spain) [37].
‘*With a stroke you*’*re finished* . . .*seen lots of my family and friends with a stroke*—*it*’*s terrible*’ (*Patient*, *UK*) [32].
**Roles in** **decision-making** **and therapy management**
*Well*, *then*, *I*’*ve already said I*’*ll accept what the doctor says. If the doctor thinks the other option is better*, *well then*, *I*’*ll keep the other option* ‘(*Patient*, *Spain*) [37].
*When I went into the* [*clinic*] *to see my doctor*, *they admitted me to the cardiac emergency*, *and they kept me there all day* … *I was in for just about a week*. … *and when I was discharged the doctors explained that they were putting me on to certain medications*, *and Coumadin was one of them* (*Patient*, *Canada*) [35].
**Satisfaction with therapy**
*I think they*’*ve been 100* %. *From my cardiologist to the family physician and to the pharmacists*, *because they*’*re just amazing*. (*Patient*, *Canada*) [35].
*I certainly get interactions with different things*, *but I haven*’*t been told* . . . [*I took*] *antibiotics for this bad flu that I had* . . . *no*, *no I hadn*’*t* [*been warned beforehand*] *and I was a bit surprised*, *but once you get the bad* [*INR*] *reading then* [*the doctor*] *says*, “*Oh yeah that was caused by such and such*”. *But you already knew that*. (*Patient*, *Australia*) [29]

Main themes are captured in bold

#### Impact of the therapy on daily living

In four studies [30, 33, 35, 37], the issue about the impact of therapy on daily living emerged. In one study [34], in which the participants had been taking warfarin, they discussed about the impact of VKAs and stated that it was small for most of them. Factors which most concerned patients were: daily management of VKAs [30, 33, 35, 37] (dietary restrictions, interactions with other drugs and alcohol consumption), monitoring, the risk of bleeding [30, 33, 35], the bruises that made them look older, limitations in certain activities such as sports, gardening or travelling [33]. Some patients stressed the changes they had made in their daily lives in order to manage VKAs [30, 33, 35]. Regarding monitoring, some patients stated that it provided them the feeling of greater control of the disease, while others reported that it made them feel calm. However, the more frequently the monitoring takes place, the greater the perception of the burden is [33] (Table 3).

#### Balance of benefits and downsides

In all the studies with patients as participants [28, 30, 32, 33, 35, 37], they discussed the benefits and downsides. The benefits that some patients associated with VKAs were: assurance of treatment success, stroke prevention [32] and a chance to live longer [28]. Patients tended to choose VKAs when they perceived a risk of stroke [28] or when they had a better understanding of the conditions associated with anticoagulation (serious and mild stroke, major bleeding, and the economic costs and disadvantages of VKAs and aspirin) [37]. In general, patients who had not taken VKAs based their opinions on experiences of family members [32, 37], friends [32], and acquaintances [37]. One study [30] showed that most patients accepted the therapy, monitoring, dose changes and compliance with the therapy. However, some patients [30] considered VKAs treatment was ineffective, and those who had not suffered a stroke or were receiving the therapy for the first time were more skeptical.

In five studies, the risks of therapy perceived by the patients were bleeding [30, 32, 33, 35, 37], hematomas [33], and other adverse effects [30]. In three studies [30, 33, 35], some patients taking VKAs explained that they had major bleeding [30, 33, 35]. Two studies [33, 35] specified that only the minority of participants had this complication. Regarding bleeding, most patients in one study [30] expressed no fear of major bleeding but others expressed anxiety [30]. In two studies some patients expressed initial fear [30, 37].

In one study [37], patients described thrombotic and hemorrhagic stroke as a complex condition with serious or irreversible effects, and they considered it was more important than major bleeding, economic costs, and the disadvantages of VKAs and aspirin. In another study, they considered that hemorrhagic stroke was a final and permanent state [32]. These perceptions were based on the experience of family members and friends [32, 37] or on their own experience [32] (Table 3).

#### Roles in decision-making and therapy management

In three studies, the patients discussed roles in decision-making and therapy management [30, 35, 37]. In one study [30], patients stated that anticoagulation was the responsibility of both physicians and patients; however, patients felt that they alone assumed the responsibility of therapy. In two studies, most patients acknowledged that decision-making was carried out by the physician only [35, 37]. This unilateral decision was related to: I) the patient’s high level of confidence in the physician experience [35], II) a paternalistic physician-patient relationship in which the patients were (also) reluctant to take an active role; and III) the idea that the professionals were trained and could be more objective [37]. In one study [37], some patients adopted the position of ignorance and delegated the decision to the professional. For a small group of patients in one study, the circumstances in which this therapy was initiated, as a medical emergency, prevented any significant patient involvement [35] (Table 3).

#### Satisfaction with therapy

Satisfaction emerged in three studies [29, 30, 35]. In one study, patients recognized that their satisfaction improved when information was given to them individually and was focused on care [30]. In another study, they expressed satisfaction with primary care staff [35]. Dissatisfaction also appeared in several studies. Patients expressed dissatisfaction with the lack of information [29, 35], the quality and level of the information provided by the family physicians [30], the difficulties and costs related to monitoring [35], and health professionals’ lack of knowledge of their medical history [35] (Table 3).

## Discussion

### Main findings

In this systematic review evaluating patients and physicians’ perceptions and attitudes towards vitamin K antagonists (VKAs), we identified several themes which could explain the underuse of VKAs. Physicians regard uncertainty in specific cases, the need of individualized decision-making, and the delegated responsibility in decision making as the main difficulties for using VKAs, while patients noted the lack of information and understanding of VKAs therapy as their main concerns.

### Our results in the context of previous research

Three themes –information to reinforce VKAs use, balance of benefits and downsides, and roles in decision-making and therapy management– were common to patients and physicians. The first two themes were closely related from the perspective of physicians. Some of them reported uncertainty regarding the balance between benefits and downsides of VKAs in cases such as polymedicated patients or in patients at high risk of falls [34, 37]. Despite the availability of guidelines and research evidence, some physicians considered that this information did not always clarify their doubts [31] in a treatment with narrow therapeutic margins [38]. They identified ambiguities in some of the guidelines, and stated that the included populations were not necessarily representative of the very elderly, the main candidates for anticoagulation [31]. The participant physicians suggested the development of individualized decision-making tools as a strategy to improve this uncertainty [37].

The information to reinforce VKAs use was also related with decision-making and therapy management roles, both of patients and physicians. Some of the physicians and most of the patients stated that the actual decision was generally carried out by physicians only [29, 35, 37], and that the information received was often inadequately provided [29] and insufficient [29, 35]. Moreover, in one study some family physicians felt that specialized physicians delegated the responsibility of decision making to them. These two sources of delegation were perceived by family physicians as a burden. To address patient’s delegation, the use at the point of care of interactive decision aids linked to guidelines could be a potential strategy [39]. The feeling of family physicians that specialized physicians delegated the responsibility of decision making to them could be explained by the lacking certainty about the treatment and the inadequate exchange of information between them.

A systematic review by Borg et al. that explored patients’ and health professionals’ experiences on VKAs therapy, raised the debate of the discrepancy in the perception that patients and health care professionals have about the decision-making models used in practice [25]. Patients’ experiences suggested a mixed of a paternalistic and interpretative model (the physician take the decision, considering the patient’s values and preferences), while some physicians stated that they practiced shared decision-making. Our systematic review also observed this discrepancy to some extent, suggesting that shared decision–making is not really taking place in clinical practice.

Knowledge and understanding of the therapy was an important issue that arose among patients only. One study included in our systematic review shows that the knowledge and understanding was worse in elderly patients than younger [30]. Given that most patients with atrial fibrillation are of advance age, to be able to make an informed decision, it is especially important that the information is provided and explained appropriately. Moreover, it is crucial to improve the quality of the information provided to patients because it is the main factor of dissatisfaction with the therapy [12, 27, 30]. Better information will improve understanding and is likely to increase the use of anticoagulation [37]. One of the factors that may explain why the difficulties with understanding of the anticoagulant treatment are greater for the elderly is that they generally are less educated [40], although this fact is changing.

Like one of our studies shows, patients tend to choose treatment with VKAs when they have a better understanding of the conditions associated with anticoagulation [37]. Therefore, understanding of the treatment with VKAs is essential for patients to assess the benefits and downsides based on their preferences [37].

A previous systematic review of quantitative studies that evaluated patients’ preferences for anticoagulants including direct oral anticoagulants (DOACs), agrees with our review that, considering the different anticoagulant treatments, patients’ preferences are based mainly on clinical aspects (reduction in the risk of stroke and moderate increase in the risk of bleeding). Nevertheless, whenever the different treatment options offer similar security and efficacy, convenience takes on importance for the decision making, such as once-a-day administration or no interactions with drugs or food. The need for monitorization of VKAs is sometimes perceived positively and sometimes negatively [41].

### Limitations and strengths

The main limitation of our study is that we cannot confirm a relationship between the factors identified and underuse of VKAs therapy [42]. Another limitation was that there were two studies which did not only included physicians and patients as participants –nurses, pharmacist, and carers participated too. Only in some cases we were able to exclude the data coming from participants other than our target groups [29, 30, 36]. Also, in one of the studies [27] it was not always possible to differentiate between patients and physicians for some of the results reported.

The review does not include studies on patients’ perceptions and attitudes towards DOACs. Nevertheless, treatment with VKAs continues to be the main group of drugs used. Additionally, a great part of the emerging deficits in the treatment with VKAs would be applicable to the treatment with DOACs, since said deficits are more related to the healthcare systems, like the lack of information provided to patients or difficulties with the coordination between primary care and hospital care.

Finally, a potential limitation is the fact that further data sources like CINAHL have not been searched. However, we believe that this may be a minor limitation since we have searched biomedical data sources with a wider and more detailed scope.

The main strength of our review is that it is the first qualitative systematic review to specifically explore factors potentially related to the underuse of oral anticoagulation in atrial fibrillation. Moreover, our review includes two more studies [36, 37] than Borg’s systematic review [25]. Another strength of our work is the research team expertise, as it includes a multidisciplinary group of experts in oral anticoagulation therapy, Evidence-Based Medicine, and qualitative research. The group also includes several authors of one of the included studies.

### Implications for practice and research

To tackle the underuse of anticoagulation there is a need to improve the quality and usability of clinical guidelines, and of the information that is provided to patients; as well as to enhance the coordination between primary care and hospital care. Linking evidence-based guidelines with decision aids could be a way forward to engage patients and physicians in shared decision-making [39]. Both guidelines and tools should be user-friendly, interactive, and based on the most rigorous evidence.

We identified some of the differences between family physicians and specialized physicians. However, further studies are needed to explore in more depth this issue. Moreover, qualitative studies evaluating the perceptions and attitudes about the direct anticoagulants should also be carried out.

## Conclusion

Physicians perceived uncertainty, need of individualized decision-making, and the feeling of delegated responsibility, as their main concerns that may be related to underuse of vitamin K antagonists, while for patients the main factors were the lack of information and understanding. Improving the quality and usability of clinical guidelines, the information provided to patients (e.g. linking decision aids and guidelines), developing tools to facilitate shared decision-making, and enhancing the coordination between primary care and hospital care could help improve the underuse of this important treatment option in patients with atrial fibrillation.

## Additional file

Additional file 1: Search strategy. (DOCX 17 kb)

## Abbreviations

DOAC: Direct oral anticoagulant
VKA: Vitamin K antagonist
