# Supplementary material for: Patients’ and physicians’ perceptions and attitudes about oral anticoagulation and atrial fibrillation: a qualitative systematic review
Source: BMC Fam Pract. 2017 Jan 13;18:3. doi: 10.1186/s12875-016-0574-0 (PMC5234257; doi:10.1186/s12875-016-0574-0)
Supplement: Additional file 1: — Search strategy. (DOCX 17 kb) [file 12875_2016_574_MOESM1_ESM.docx]

**Additional file 1: Search strategy**

Literature search strategy

**PubMed**

1 value*[tiab] (1206162)

2 preference*[tiab] (86251)

3 attitude*[tiab] (90673)

4 Perception*[tiab] (136398)

5 perspective*[tiab] (162405)

6 knowledge*[tiab] (368454)

7 decision making [tiab] (60748)

8 "Health Knowledge, Attitudes, Practice"[MeSH] (65212)

9 "Attitude of Health Personnel"[MeSH] (113349)

10 "Attitude to Health"[MeSH] (270258)

11 "Physician's Practice Patterns"[MeSH] (37559)

12 1 or 2 or 3 or 4 or 5 or 6 or 7 or 8 or 9 or 10 or 11 (2231336)

13 "Anticoagulants"[MeSH] (53430)

14 anticoagulant*[tiab] (39783)

15 anticoagulat*[tiab] (25432)

16 13 or 14 or 15 (87571)

17 Atrial fibrillation[MeSH] (31180)

18 atrial fibrillation[tiab] (35419)

19 17 or 18 (43710)

20 12 and 16 and 19 (1062)

**EMBASE**

1 value*.ti,ab. (1626713)

2 preference*.ti,ab. (104388)

3 attitude*.ti,ab. (118190)

4 perception*.ti,ab. (166009)

5 perspective*.ti,ab. (199203)

6 knowledge.ti. (43053)

7 decision making.ti. (16017)

8 physician attitude/ (39748)

9 exp attitude to health/ (75592)

10 1 or 2 or 3 or 4 or 5 or 6 or 7 or 8 or 9 (2259548)

11 exp anticoagulant agent/ (484083)

12 anticoagulant*.ti,ab. (58533)

13 anticoagulat*.ti,ab. (36970)

14 11 or 12 or 13 (506196)

15 exp heart atrium fibrillation/ (71859)

16 atrial fibrillation.ti,ab. (54330)

17 15 or 16 (78687)

18 10 and 14 and 17 (1844)

19 phenomenol$.af. (19400)

20 qualitative stud$.mp. (17225)

21 nursing methodology research.mp. (13236)

22 focus group$.mp. (22374)

23 discourse analysis.mp. (1296)

24 content analysis.mp. (12442)

25 ethnograph$.mp. (6548)

26 ethnological research.mp. (12)

27 purposive sample.mp. (1769)

28 observational method$.mp. (1369)

29 field stud$.mp. (13058)

30 theoretical sampl$.mp. (388)

31 phenomenology/ (6443)

32 phenomenological research.mp. (278)

33 life experience$.mp. (3642)

34 grounded theory.mp. (6081)

35 (emic or etic or hermeneutic$ or heuristic$ or semiotic$).af. or (data adj1 saturat$).tw. or participant observ$.tw. (14745)

36 (social construct$ or (postmodern$ or post-structural$) or (post structural$ or poststructural$) or post modern$ or post-modern$ or feminis$ or interpret$).mp. (348715)

37 (field adj (study or studies or research)).tw. (13282)

38 purposive sampl$.af. (3005)

39 theoretical sampl$.af. (388)

40 ((purpos$ adj4 sampl$) or (focus adj group$)).af. (28544)

41 (account or accounts or unstructured or open-ended or open ended or text$ or narrative$).mp. (484267)

42 focus group$.af. (22440)

43 content analysis.af. (12442)

44 thematic analysis.af. (4398)

45 constant comparative.af. (1527)

46 discourse analys?s.af. (1323)

47 ((discourse$ or discurs$) adj3 analys?s).tw. (1247)

48 (constant adj (comparative or comparison)).af. (2251)

49 narrative analys?s.af. (530)

50 19 or 20 or 21 or 22 or 23 or 24 or 25 or 26 or 27 or 28 or 29 or 30 or 31 or 32 or 33 or 34 or 35 or 36 or 37 or 38 or 39 or 40 or 41 or 42 or 43 or 44 or 45 or 46 or 47 or 48 or 49 (913198)

51 18 and 50 (110)

**PsycINFO**

1 value*.ti,ab. (146217)

2 preference*.ti,ab. (48614)

3 attitude*.ti,ab. (100125)

4 perception*.ti.ab. (149972)

5 perspective*.ti,ab. (153523)

6 knowledge.ti. (23092)

7 decision making.ti. (11522)

8 exp Health Personnel Attitudes/ (14634)

9 1 or 2 or 3 or 4 or 5 or 6 or 7 or 8 (549795)

10 exp Anticoagulant Drugs/ (291)

11 anticoagulant*.mp. (452)

12 anticoagulat*.mp. (329)

13 10 or 11 or 12 (738)

14 exp "Fibrillation (Heart)"/ (312)

15 Atrial fibrillation.mp. (544)

16 14 or 15 (631)

17 9 and 13 and 16 (19)

**ISI WoK**

1 Topic=(value* OR preference* OR attitude* OR perception* OR perspective* OR knowledge) (>100,000)

2 Topic=(phenomenol* or qualitative stud* or qualitative or focus group or discourse analysis or content analysis or ethnograph* or purposive sample OR theoretical sampl* or field stud* or phenomenolog* or grounded theory or hermeneutic* or heuristic* or participant observ* or social construct* or narrative* or open ended or field stud* or discourse analys*) (>100,000)

3 Topic=(anticoagulant*) (74,511)

4 Topic=(anticoagulat*) (25,952)

5 4 or 3 (88,161)

6 Topic=(atrial fibrillation) (48,809)

7 6 and 5 and 1 (872)

8 7 and 2 (17)
